# First complete genome sequence of infectious laryngotracheitis virus

**DOI:** 10.1186/1471-2164-12-197

**Published:** 2011-04-19

**Authors:** Sang-Won Lee, Philip F Markham, John F Markham, Ivonne Petermann, Amir H Noormohammadi, Glenn F Browning, Nino P Ficorilli, Carol A Hartley, Joanne M Devlin

**Affiliations:** 1 Asia-Pacific Centre for Animal Health, School of Veterinary Science, The University of Melbourne, Parkville, Victoria, 3010, Australia; 2 Applied Biosystems, Australia; 3 National ICT Australia, Department of Electrical and Electronic Engineering, The University of Melbourne, Victoria, 3010 Australia

## Abstract

**Background:**

Infectious laryngotracheitis virus (ILTV) is an alphaherpesvirus that causes acute respiratory disease in chickens worldwide. To date, only one complete genomic sequence of ILTV has been reported. This sequence was generated by concatenating partial sequences from six different ILTV strains. Thus, the full genomic sequence of a single (individual) strain of ILTV has not been determined previously. This study aimed to use high throughput sequencing technology to determine the complete genomic sequence of a live attenuated vaccine strain of ILTV.

**Results:**

The complete genomic sequence of the Serva vaccine strain of ILTV was determined, annotated and compared to the concatenated ILTV reference sequence. The genome size of the Serva strain was 152,628 bp, with a G + C content of 48%. A total of 80 predicted open reading frames were identified. The Serva strain had 96.5% DNA sequence identity with the concatenated ILTV sequence. Notably, the concatenated ILTV sequence was found to lack four large regions of sequence, including 528 bp and 594 bp of sequence in the UL29 and UL36 genes, respectively, and two copies of a 1,563 bp sequence in the repeat regions. Considerable differences in the size of the predicted translation products of 4 other genes (UL54, UL30, UL37 and UL38) were also identified. More than 530 single-nucleotide polymorphisms (SNPs) were identified. Most SNPs were located within three genomic regions, corresponding to sequence from the SA-2 ILTV vaccine strain in the concatenated ILTV sequence.

**Conclusions:**

This is the first complete genomic sequence of an individual ILTV strain. This sequence will facilitate future comparative genomic studies of ILTV by providing an appropriate reference sequence for the sequence analysis of other ILTV strains.

## Background

Infectious laryngotracheitis virus (ILTV) is an alphaherpesvirus that causes acute respiratory disease in chickens. This disease causes economic loss in poultry industries worldwide and is a significant concern for animal health and welfare [1]. The virus contains a linear, double-stranded DNA genome in a herpesvirus type D arrangement. This genome arrangement consists of a unique long region and a unique short region flanked by identical internal and terminal repeat sequences [2,3], with the short region able to invert with respect to the long region. In previous studies, several regions of the ILTV genome of different strains have been sequenced and annotated [3-12]. Recently, a full genomic sequence of ILTV was assembled by concatenating partial sequences of six different ILTV strains [13]. However, the whole genomic sequence of a single strain of ILTV has not been reported. In this study, the whole genome sequence of a commercial live attenuated vaccine strain of ILTV was examined using high-throughput sequencing technology.

## Results and Discussion

### Sequencing and coverage

Genomic sequencing using the SOLiD™ system generated 526.69 Mb of sequence and 12,046,726 reads. A total of 230 contigs were mapped to the virus genome after *de novo *assembly. A consensus sequence of 137,693 bp (without the terminal repeat region) was generated after assembly of contigs using the concatenated reference sequence. The depth of coverage against the concatenated ILTV sequence was greater than 150-fold. This depth of coverage exceeds the level required for precision whole-genome sequencing and demonstrates the suitability of the massively parallel, ligation-mediated sequencing method for herpesvirus genome sequencing [14]. Although the depth of coverage was sufficient to generate long contigs able to cover the whole genome, a total of 185 gaps or regions of ambiguous sequence were detected after *de novo *assembly. Most gaps were less than 100 bp in size. While sonication theoretically produces random fragment libraries, some sequences, such as A-T rich regions, have been found to be more susceptible to breakage [15]. Such weak regions may have been cleaved more frequently during our fragment library preparation, resulting in an absence of high-throughput sequencing data across these regions.

### Overview of the Serva ILTV genome

**Figure 1:**
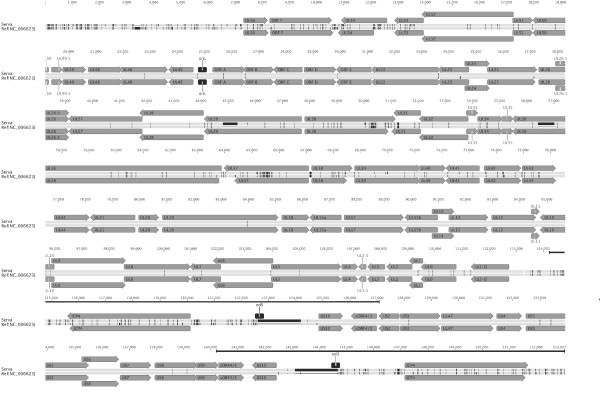
**Nucleotide alignment of the Serva ILTV sequence and the concatenated ILTV reference sequence**. Single vertical black lines indicate single nucleotide differences. Wider black boxes indicate larger regions of sequence differences, including areas where there are large gaps. Dashes indicate single base gaps in sequence alignments. The alignment was performed using Clustal W version 2.0. The locations and sizes of predicted ORFs and origins of DNA replication were annotated using the Geneious software package. The solid lines below the residue numbers indicate the locations of the internal and terminal repeat sequences.

The general features of the Serva ILTV genome sequence were examined and were consistent with those described in previous genome studies of ILTV [3-12]. The genome size of the Serva ILTV strain was 152,630 bp, with a G + C content of 48%. The unique long and unique short sequences were 113,930 bp and 13,094 bp in length, respectively, with the unique short sequence flanked by internal and terminal repeat sequences (each 12,803 bp in length). A total of 80 predicted open reading frames (ORFs) were identified. Sixty-five ORFs were located within the long unique region, nine within the unique short region, and three pairs of ORFs within the repeat regions of the genome. An annotated genome map of the Serva ILTV strain is shown in Figure 1.

### Comparison of the Serva ILTV genome with the concatenated ILTV reference sequence

Comparative analyses showed that the Serva strain had the same gene arrangement as that of the concatenated ILTV genomic reference sequence (Figure 1). In complete genomic alignment analysis, the Serva strain had 96.5% DNA sequence identity with the concatenated sequence. Differences between the two sequences were mostly located within the left terminal region of the genome (extending over 18,169 bp), the middle of the unique long region of the genome (extending over 31,332 bp) and within the repeat regions (extending over 8,364 bp) (Figure 1). In the concatenated reference sequence, all these regions were obtained from SA-2 ILTV, a commercial vaccine strain of ILTV produced from an Australian field isolate [13]. Australian strains of ILTV may contain different genetic features compared with other ILTV strains, due to their evolution in a geographically isolated environment [16]. Excluding SA-2 sequence regions, DNA sequence identity between the Serva strain sequenced in this study and the concatenated sequence was 99.9%, containing 41 single-nucleotide polymorphisms and 13 nucleotide insertions or deletions. This high level of identity is consistent with the stable genome and low mutation rates observed in herpesviruses [17,18]. All nucleotide differences between the concatenated reference and the Serva strain of ILTV are listed in Additional File 1.

**Figure 2:**
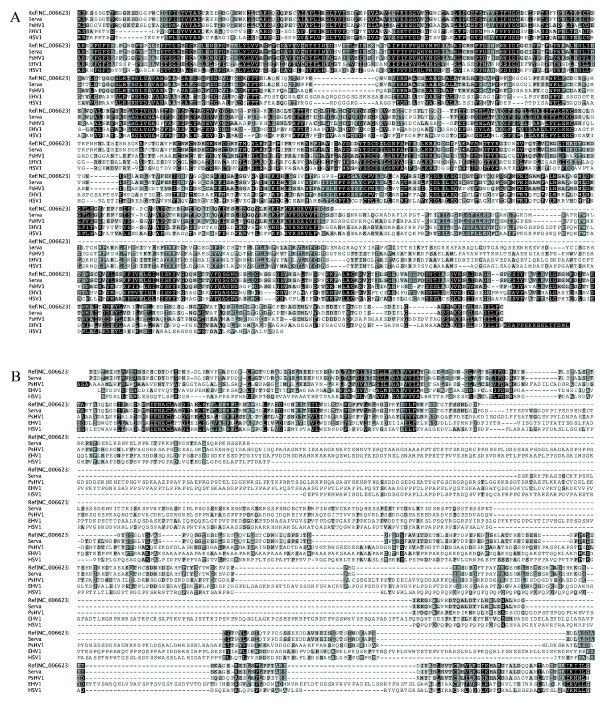
**Amino acid alignment of predicted translation products of homologous genes from the Serva ILTV strain, the concatenated ILTV reference sequence and other alphaherpesviruses**. (A) UL29 gene product. (B) C terminal part of the UL36 gene product. Black boxes indicate 100% identity, dark grey boxes indicate 80-99% identity and pale grey boxes indicate 60-79% identity. Dashes indicate single amino acid gaps in sequence alignments. Alignments were performed using Clustal W version 2.0. The Blosum62 matrix was used to score sequence alignments.

Four large regions of sequence were absent in the concatenated ILTV sequence compared with the Serva strain. These included 528 bp and 594 bp of sequence missing in the UL29 and UL36 genes, respectively. Additionally, in each of the repeat regions the concatenated sequence lacked 1,563 bp of sequence compared to the Serva strain. To verify these findings, PCR was used to amplify the relevant regions of the UL29 and UL36 genes, and the repeat region of the Serva genome. The PCR products were sequenced using Sanger sequencing. The results obtained from Sanger sequencing were identical to those obtained by the high-throughput sequencing. The predicted translation products of the UL29 and UL36 genes of the Serva strain more closely resembled, and had more conserved domains when compared with, the UL29 and UL36 homologues from other alphaherpesviruses than the predicted products derived from the concatenated genome sequence (Figure 2). These results support the conclusion that the newly determined additional sequences are genomic sequence of ILTV, not sequencing errors. Within the left terminal region of the genome, the Serva sequence had a 189 bp deletion compared to the concatenated sequence (Figure 1).

There were differences in size of more than 50 amino acids between the predicted translation products of the UL54, UL29, UL30, UL36, UL37 and UL38 genes in the concatenated reference sequence and the Serva sequence. Smaller differences in the predicted sizes of other gene products were also identified (Table 1). All amino acid differences between predicted translation products in the concatenated reference sequence and the Serva sequence are listed in Additional File 2.

**Table 1:** Differences in the size of predicted translation products between the Serva ILTV strain and the concatenated ILTV reference sequence

ORF	**Concatenated ILTV reference sequence**^**a**^			**Serva ILTV sequence**^**a**^		
		
	Nucleotide start	Nucleotide end	ORF length (aa)	Nucleotide start	Nucleotide end	ORF length(aa)
UL56	7316	8170	855 (284)	7062	7886	825 (274)
ORF F	8301	10559	2259 (752)	8017	10257	2241 (746)
**UL54**	**10784**	**12046**	**1263 (420)**	**10646**	**12280**	**1635 (544)**
UL25	35392	37110	1719 (572)	35149	36927	1779 (592)
**UL29**	**44095**	**47094**	**3000 (999)**	**43851**	**47381**	**3531 (1176)**
**UL30**	**47271**	**50294**	**3024 (1007)**	**47558**	**50836**	**3279 (1092)**
UL31	50464	51483	1020 (339)	50826	51770	945 (314)
**UL36**	**54914**	**62584**	**7671 (2556)**	**55201**	**63555**	**8355 (2784)**
**UL37**	**63209**	**65881**	**2673 (890)**	**63694**	**66762**	**3069 (1022)**
**UL38**	**66045**	**67283**	**1239 (412)**	**66926**	**68374**	**1449 (482)**
UL42	72398	73696	1299 (432)	73276	74586	1311 (436)
ICP4	114497	118888	4392 (1463)	115301	119749	4449 (1482)
	142837	147228	4392 (1463)	146812	151260	4449 (1482)

^a ^Bold texts indicate large differences (>50 amino acids) in the size of predicted gene products

Given the conserved nature of the ILTV genome, future studies examining the genomic variation between different strains of ILTV may represent a strategic approach to examining the molecular pathogenesis of this virus. In particular it would be useful to compare sequence differences between virulent and attenuated strains of ILTV, especially within genes already known to be associated with ILTV virulence including gC, UL0, gG, gJ and TK genes [19-23].

## Conclusions

This is the first complete genomic sequence of an individual ILTV strain. A number of differences between this strain and the concatenated reference ILTV sequence were identified. Significantly, four large missing regions were identified in the published concatenated reference sequence. Missing regions of genomic sequence considerably hamper genomic assembly, so the Serva sequence assembled in this study represents a much improved reference sequence for future high throughput ILTV sequencing studies and comparative genomic analyses.

## Methods

### Virus strain

This study utilised the chicken embryo origin live attenuated Serva vaccine strain of ILTV (Nobilis^® ^ILT, Intervet), which has been recently introduced into Australia [16]. Virions were purified by Ficoll gradient centrifugation directly from commercial vaccine vials. Pelleted virions were washed and resuspended in TNE buffer (0.01 M Tris, 0.2 M NaCl, 1 mM EDTA, pH 7.4).

### High-throughput sequencing

Following purification, total viral genomic DNA was extracted using the High Pure PCR Template Preparation Kit (Roche). Sequencing was performed using parallel, ligation-mediated sequencing technology (SOLiD™ 3 system, Applied Biosystems) following the manufacturer's standard procedures. Briefly, 1 μg of ILTV DNA was sheared and P1 and P2 adaptors were ligated to the fragments. Ligated DNA fragments were size-selected to an average length of 170 bp and amplified for 10 cycles. Approximately 10 pg of this ILTV fragment library/μl, as well as 50 pg of a similarly generated library of an unrelated bacterial genome of around one megabase pairs/μl, were added to an emulsion with 80 million beads. The libraries were sequenced in parallel using a flow cell divided into 8 segments. The resulting reads were unambiguously mapped to either the viral or the bacterial genome, allowing up to two mismatches for each read. The software package Velvet version 0.7.55 was used to perform *de novo *assembly of all reads [24] and the resulting contigs for the virus were identified bioinformatically and then aligned to the complete concatenated ILTV genomic sequence, with the exception of the terminal repeat region (identical to the internal repeat region), which was excluded from the analysis.

### ILTV DNA sequence analysis

The software package Geneious [25] was used to manually curate the alignments of the contigs and to produce a consensus sequence with reference to the original mapped reads. Any sequence gaps or ambiguous regions of sequence were amplified and sequenced by Sanger sequencing methods using BDT version 3.1 (Applied Biosystems). Nucleotide and amino acid sequence alignments were performed using ClustalW version 2.0 [26], ORFs were then annotated using the Geneious software package. Open reading frames containing more than 50 amino acids were predicted using the ORF finder function of Geneious, based on the complete concatenated ILTV genomic sequence.

### Nucleotide sequence accession numbers

The complete genome sequence of Serva ILTV has been deposited in the NCBI GenBank database under accession HQ_630064. The concatenated ILTV genomic sequence is available in the GenBank database under accession NC_006623. Nucleotide sequences for psittacid herpesvirus-1 (PsHV1), equid herpesvirus-1 (EHV1) and herpes simplex virus-1 (HSV1) were also utilized in this study. The translated amino acid sequences for the UL29 genes of these viruses are available in the GenBank database under accessions NP_944402, YP_053076 and NP_044631, respectively. The translated amino acid sequences for the UL36 genes of these viruses are available in the GenBank database under accessions NP_944409, YP_053069 and ABI63498, respectively.

## Authors' contributions

S-WL carried out the sequencing studies, sequence analyses, sequence alignments and drafted the manuscript. NPF performed preparation of viral DNA. IP performed sequencing. JFM performed sequence analyses. CAH performed preparation of viral DNA, participated in sequence analyses and helped to draft the manuscript. JMD, PFM, GFB and AHN conceived of the study, designed and coordinated the study, participated in preparation of viral DNA, sequencing studies, sequence analyses, and helped to draft the manuscript. All authors read and approved the final manuscript.

## Supplementary Material

Additional file 1 **Summary of nucleotide differences**. Nucleotide differences detected between the concatenated ILTV reference sequence and the Serva ILTV sequence after whole genome alignment. Click here for file

Additional file 2 **Summary of all amino acid differences**. Amino acid differences detected between the predicted translation products of the concatenated ILTV reference sequence and the Serva ILTV sequence. Click here for file
